# Neural Correlates of Temporal Credit Assignment in the Parietal Lobe

**DOI:** 10.1371/journal.pone.0088725

**Published:** 2014-02-11

**Authors:** Timothy M. Gersch, Nicholas C. Foley, Ian Eisenberg, Jacqueline Gottlieb

**Affiliations:** 1 Department of Neuroscience, Columbia University, New York, New York, United States of America; 2 The Kavli Institute for Brain Science Columbia University, New York, New York, United States of America; University of Sheffield, United Kingdom

## Abstract

Empirical studies of decision making have typically assumed that value learning is governed by time, such that a reward prediction error arising at a specific time triggers temporally-discounted learning for all preceding actions. However, in natural behavior, goals must be acquired through multiple actions, and each action can have different significance for the final outcome. As is recognized in computational research, carrying out multi-step actions requires the use of credit assignment mechanisms that focus learning on specific steps, but little is known about the neural correlates of these mechanisms. To investigate this question we recorded neurons in the monkey lateral intraparietal area (LIP) during a serial decision task where two consecutive eye movement decisions led to a final reward. The underlying decision trees were structured such that the two decisions had different relationships with the final reward, and the optimal strategy was to learn based on the final reward at one of the steps (the “F” step) but ignore changes in this reward at the remaining step (the “I” step). In two distinct contexts, the F step was either the first or the second in the sequence, controlling for effects of temporal discounting. We show that LIP neurons had the strongest value learning and strongest post-decision responses during the transition after the F step regardless of the serial position of this step. Thus, the neurons encode correlates of temporal credit assignment mechanisms that allocate learning to specific steps independently of temporal discounting.

## Introduction

Converging evidence from recent research is consistent with the idea that animals solve simple decision tasks in a manner consistent with basic reinforcement learning mechanisms, and that variables related to these mechanisms are encoded in individual cells [1], [2]. Specifically, sensorimotor cells in cortical and subcortical structures are thought to encode the values of competing options, and to update these values based on of reward prediction errors in midbrain dopamine cells [2], [3]. An intensively investigated value representation is found in the lateral intraparietal area (LIP), a cortical area involved in target selection for spatial attention and eye movement control [4]. LIP neurons have visual-spatial receptive fields (RF) and respond selectively for task-relevant targets relative to distractors, and their responses scale with target value, whether value is manipulated through the probability or magnitude of an expected reward [5], [6], the delay to the future reward [7], or the relative values of the alternative options [8]. Thus, LIP cells have been proposed to act as an intermediate decision stage that encodes action values and provides input to a final step of action selection [2], [3].

The decision studies carried out so far have been largely limited to simple paradigms where animals choose individual actions and receive discrete feedback regarding each action. However, in natural behavior achieving a goal typically requires *sequences* of action that may be extended in space and in time. Moreover, each of the actions in the sequence may have a different significance for the final goal [1], [9], [10]. Imagine, for example, that you are an athlete trying to improve your sprint time at a daily practice. In this case you will want to pay attention to your choice of stretching regimen and learn about the value of that regimen based on your sprint performance at today’s practice. However, you would ideally *not* update the value of your decision to wear a red rather than a blue shirt. Even though this decision is a valuable part of the action chain and may be closer in time to your final goal, it has little bearing on your sprint time. In these more complex conditions, therefore, animals require *credit assignment* mechanisms that can link a global reward with the significant steps based on task-specific or contextual information.

Although the importance of credit assignment mechanisms is widely recognized in computational research, their neural correlates remain almost entirely unexplored. In computational models, credit assignment is implemented directly by increasing learning rates at specific steps [10] or indirectly, by using eligibility traces to prolong the memory for a recent action and increase its eligibility for a later reinforcement [11]. Studies of decision making, in contrast, have explained the results of simple single-step tasks using algorithms where learning depends solely on time – i.e., reward prediction errors from a final reward propagate automatically to all preceding actions subject only to temporal discounting (e.g., [12], [13]). Likewise, studies of sequential actions have focused on memory and ordering processes but not on credit assignment mechanisms (e.g., [14], [15]).

In the present study, we sought to identify the neural correlates of credit assignment by recording single neuron responses in area LIP during a sequential task where two eye movement decisions were required to obtain a final reward. The critical manipulation was that, in two distinct contexts, either the first or the second decision was important for the final reward. In contrast, the choice at the remaining step could not change the size of the final reward and would optimally be based on the immediate reward. We refer to these two decisions as the “F” and the “I” steps, to indicate that they are optimally based on, respectively, the final or immediate rewards. We show that the monkeys adopted an optimal strategy, learning selectively based on the final reward at the “F” but not at the “I” step. This selective learning was seen whether the F step was the first or second in the sequence, showing that it was independent of temporal discounting. LIP cells encoded this strategy by showing stronger value learning and enhanced post-decision responses after the F relative to the I step. Thus, the cells encode credit assignment mechanisms consisting of elevated learning rates and memory (eligibility) traces that highlight specific, significant steps in a sequence.

## Results

### Task design

**Figure 1:**
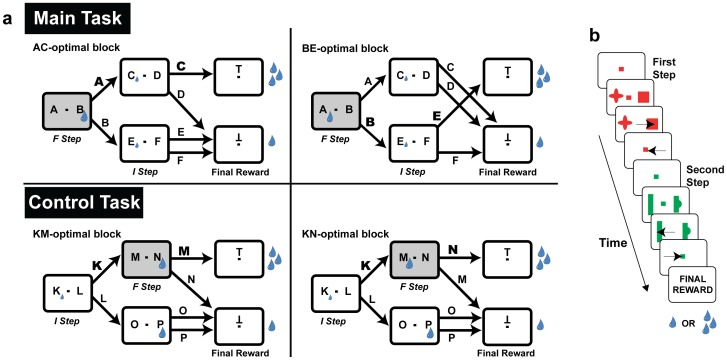
Behavioral task. (a) Transition contingencies for the main task and the control task. Each target is denoted by a letter and drops indicate rewards. Optimal choices are denoted in bold. (b) The sensorimotor events on a representative trial. In each trial the monkeys made two eye movement decisions to obtain a reward. After initiating a trial by acquiring fixation the monkeys were shown the first pair of targets (randomly placed on either side of fixation) and, after a brief delay, chose one target by making a saccade to it. The monkeys then returned to fixation, were shown the second pair of targets and made their second choice. The trial ended with a final return to fixation and delivery of the final reward. The target sequence that delivered the largest reward was switched across trial blocks and the monkeys discovered this sequence by trial and error.

Two monkeys completed a sequential decision task where they made two eye movement decisions to obtain a final reward. In each trial the monkeys began by achieving central fixation, after which they were shown the first pair of targets and chose one by making a saccade to it (**Fig. 1b**). The monkeys then returned to fixation, were shown the second target pair and made their second choice. After a final return to fixation the trial ended with the delivery of a large or small final reward (see *Methods* for additional details). The task was run in separate blocks of ∼150 trials, such that in each block a randomly selected choice sequence led to the largest reward, and the monkeys had to discover this sequence by trial and error. Our focus was on the monkeys’ exploratory strategy – the ways in which they sampled the alternative options to find the optimal path.

The key task manipulation was that, in two different contexts, the optimal strategy was to explore selectively based on the final reward *only* at the first or *only* at the second step, and ignore changes in the final reward at the remaining step. We refer to the step where it was optimal to *explore* as the “F” step to indicate the strong relationship between this step and the final reward. We refer to the step where it was optimal to *withhold exploration* as the “I” step, to indicate the closer relation between this step and the immediate reward (see below). As we show below, if the monkeys adopted a step and context-specific exploration strategy this would be evidence that they can assign credit for a global reward to specific steps. In contrast, if they adopted a less efficient undifferentiated strategy, this would indicate an absence of credit assignment mechanism.

We established the significance of a decision step by manipulating the transition contingencies leading to the final reward, resulting in four different decision trees. These decision trees, which were hidden from the monkeys, are reproduced for the reader in **Fig. 1a** using abstract notation where each target is denoted by a letter. In the main task context (signaled by the use of a fixed complement of saccade targets, A-F in **Fig. 1a**, top) the F step was the initial step in the sequence. In any given trial block, the monkeys had to discover whether the largest final reward was reached by selecting either target A or B at this step (e.g., whether they were in an AC-optimal or BE-optimal configuration; **Fig. 1a**, top). At the second step, by contrast, the optimal choice was determined by the immediate reward (see below) and *always* consisted of targets C or E (in, respectively, an AC-optimal, or a BE-optimal block). Thus, in this task context, the optimal strategy when sensing a change in the final reward was to evaluate the final reward associated with the alternative options at the first, F, step, but keep a fixed preference for one of the options at the second, I, step. The control task was signaled by a distinct target set (**Fig. 1a**, bottom, targets K-P), and was similar in all respects except that the F step was the second in the sequence. At this step, targets M or N could lead to the large final reward in different trial blocks, while at the first step, the optimal choice consisted of target K in all trial blocks.

In sum, the decision task had a hierarchical structure. Upon entering a trial block the monkeys could infer the context based on the saccade target set, but had to search for the path leading to the largest reward. Our question was whether their search was focused on a specific step in context-dependent fashion. It is important to note that such a difference was in no way dictated by the task contingencies or training regimen. Even though the appearance of the targets signaled a difference between the two contexts, it did not instruct the monkeys that one step was more significant, or which was the significant step. Any physical sequence of events seen by the monkey (e.g., **Fig. 1b**) was equally consistent with the first or the second step being more significant, or with an undifferentiated strategy where the search extended to both decision steps. Thus, if the monkeys implemented context specific exploration, this would indicate that they had *inferred* aspects of the task structures.

We implemented a final manipulation where we provided small immediate rewards after each decision step, in order to underscore the step-specific differences and facilitate the interpretation of the data. Because the immediate rewards were very small, behavior was ultimately governed by the final reward (see *Methods* for specific values and the results below). However, by selectively assigning the immediate rewards we could constrain the interpretation of the monkeys’ choices. At the F step in each context, the immediate reward was given for the target that was *not* optimal in the long run (e.g., target B in an AC-optimal block and target A in a BE-optimal block). Therefore, at the F step the optimal strategy was to suppress the pull of the immediate reward and learn target values based on the final reward, whether this step was the first or second in the sequence (main or control contexts). At the I step, in contrast, the immediate reward was assigned to a fixed target and the optimal strategy was to choose based on this reward. For example, target C in the main task was optimal whether the final reward associated with it was large (e.g., in an AC-optimal block) or small (e.g., in a BE-optimal block). Thus, the optimal strategy at this step was to ignore the final reward and choose solely based on the immediate reward. Such a strategy, which weights the final reward strongly or weakly depending on context regardless of its temporal proximity to the action selection, clearly distinguishes a selective from a temporally discounted learning rule.

### Behavior

**Figure 2:**
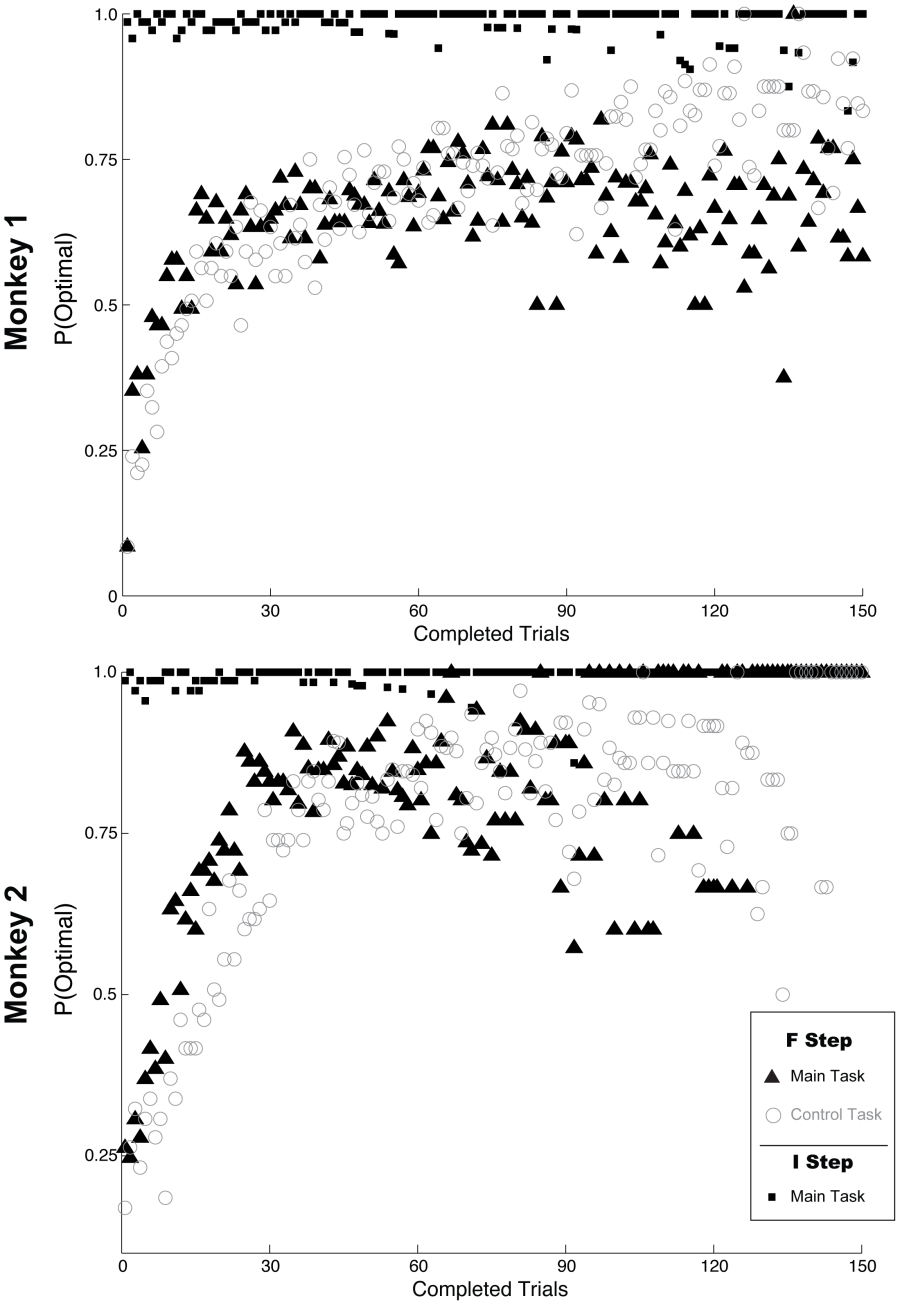
Behavior. The filled symbols show the fraction of choices of the optimal target at the first and second steps for the main task, and at the second step for the control task. Each point represents the average across sessions for a given trial number in a block.

The monkeys modulated their exploration in context-specific fashion, modifying their choices selectively at the F step but maintaining a stereotyped preference at the I step (**Fig. 2**). At the F step in both tasks, both monkeys started out a trial block with a bias toward the *non*-optimal target (which received the larger immediate reward) and slowly reversed this bias, reaching an asymptote of 75–80% optimal choices geared toward the large final reward. Learning was slow, consistent with a trial and error mechanism. A behavioral learn-point (defined as the last trial of 7 consecutive optimal choices) was reached, on average, after 41 trials in the main task and 50 trials in the control task (two-tailed t-test, p > 0.05 between tasks; monkey 2, learn points 25 (+/–5) for main vs 44 (+/–3) for control, p<0.05; monkey 1: learn points 55 (+/–5) for main, 54 (+/–4) for control, p > 0.05). This slow learning was not due to spurious factors such as incomplete training on the task (linear regression, p > 0.1 for effect of session number for each monkey and task) or an idiosyncratic bias for a spatial location (p > 0.05 for each monkey and task). Moreover, gradual learning was seen in each individual session with no evidence of a step-like switch to the optimal path, showing that it was not an artifact of averaging across sessions (**Fig. S1**). Therefore, even though the monkeys experienced only two alternative paths in each context, they seemed to re-discover the optimal one *de novo* by trial and error, consistent with previous reports [16], [17].

In contrast with this robust learning at the F step, changes in the final reward had no effect at the I step. In the control task, the monkeys chose the immediately rewarded target on 100% of the trials (for clarity, these data are omitted from **Fig. 2** **)**, and, in the main task they chose this target on 98.5% of trials even though this choice was temporally proximal to the final reward (**Fig. 2**, black squares; 11,298/11,465 trials across all recording sessions). As we show below, this selective learning differs from a reinforcement mechanism devoid of credit assignment, which would produce obligatory exploration based on the final reward at the I step, suggesting that the monkeys appropriately assigned credit to the significant step in context dependent fashion.

### LIP neurons encode relative target values and respond differently at the F and I steps

To examine the neural correlates of this differentiated strategy, we recorded the activity of 96 neurons (52 in monkey 1) that were identified as belonging to LIP based on their location in the intraparietal sulcus and spatially selective delay period activity on a memory guided saccade task (see *Methods* and **Fig. S2**). We placed the saccade targets inside and opposite a neuron’s RF and examined responses to target selection as an index into internal valuation.

LIP neurons are known to carry two multiplexed signals, a primary response encoding the saccade direction, and a modulation of this response by expected reward [6]. To disambiguate these factors we used the fact that, at the F, step the monkeys made both optimal and non-optimal choices, and the locations of optimal target could fall inside or opposite the RF (see *Methods*), statistically dissociating value from saccade direction. Therefore, at the F step we fit each neuron’s firing rates with the bivariate regression:
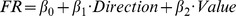
Here FR are firing rates in a sliding window aligned on target and saccade onset (50 ms window, 1 ms step), β*_0_,* β*_1_,* and β*_2_* are fitted coefficients, and direction and value were coded as dummy variables of 0 or 1. In this analysis a positive direction coefficient indicates higher firing for a saccade toward the RF, and a positive value coefficient indicates higher firing when the optimal target was in the RF regardless of saccade direction. (Note that, although we can assess the significance of each coefficient, the absolute magnitude of the value and direction coefficients depend on the arbitrarily chosen dummy variables and cannot be meaningfully compared to each other.)

**Figure 3:**
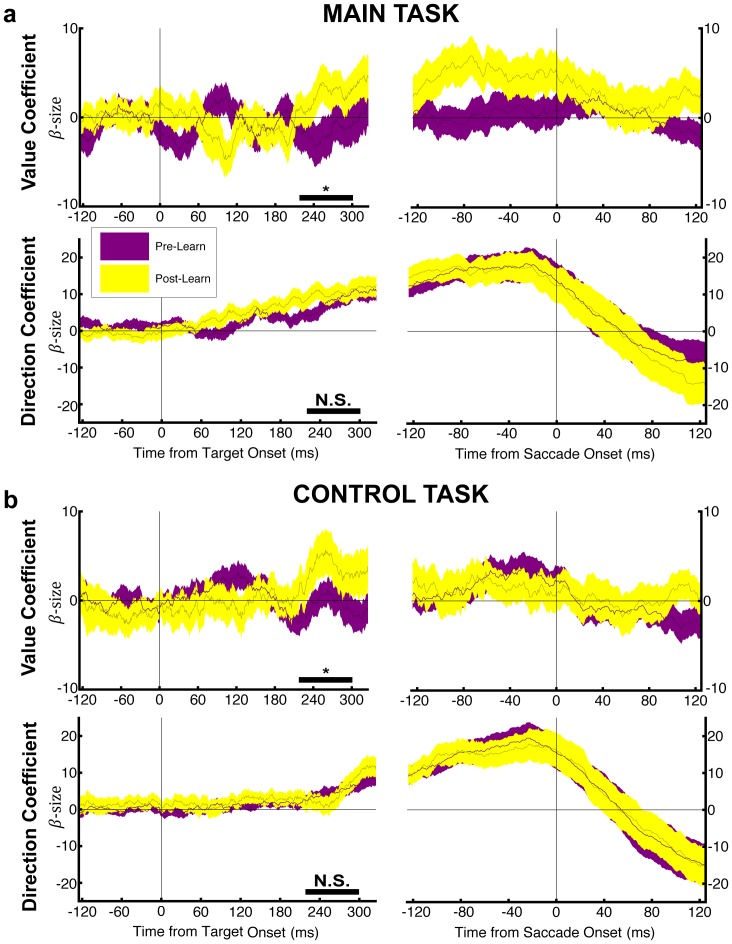
LIP neurons encode value independently of saccade direction. (a) Value and direction coefficients in the main task. The top and bottom panels show the time course of, respectively, the value and direction signals in the LIP response, aligned on target onset on the left, and saccade onset on the right. Traces show mean and SEM. The horizontal bars show paired comparisons of pre- and post-learning coefficients in the 200–300 ms after target onset (stars, p<0.05). (b) Value and direction coefficients in the control task. Same format as in (a).

LIP neurons showed significant positive coefficients for value and saccade direction in both the main and the control tasks; **Fig. 3**). The value coefficient peaked earlier during the decision period and was higher in trials that followed rather than preceding the learning point (200–300 ms, stars, p<0.05). In contrast, the direction coefficient peaked later during the pre-saccadic epoch and did not show significant learning effects at any time during the decision interval. The pattern found in the full data set was replicated individually in each monkey (**Fig. S3**) and, in individual cells, 59/96 neurons had significant value coefficients in at least one task (criterion of at least one time bin significant at p<0.001; 36/96 cells for the main task; 38/96 cells for the control task). We found no correlation between the value coefficient and the fraction of choices on a session-by-session basis for optimal (r  =  0.04) or non-optimal choices (r  =  0.12). This supports the prevailing view that the cells encode an intermediate stage of learning and valuation that influences, but is not rigidly mapped onto the final choice [3], [18].

Note that the above regression analysis, which separates value from saccade direction, could not be performed on the I step because the monkeys never selected the non-optimal target at this step. However, to compare neural responses at the F and I steps we calculated responses to target selection, defined as the firing rate difference between trials in which the saccade was directed toward and opposite the RF (or equivalently, trials in which the neurons responded to a target or a distractor in its RF). Target selection reflects the combined effects of direction and value and could reveal difference in computations between the two steps.

**Figure 4:**
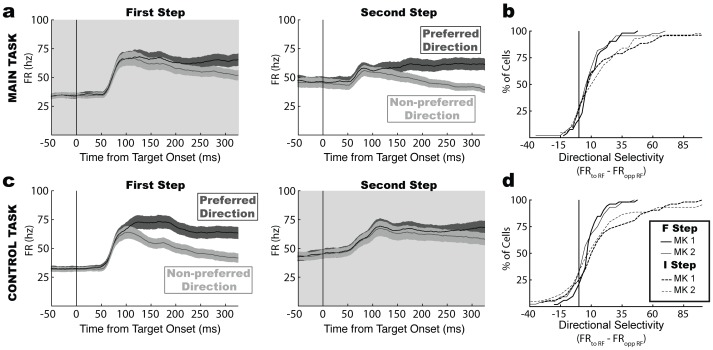
LIP target selection responses are weaker at the F relative to the I step. (a) Average firing rate on completed trials in the main task (mean and SEM in 96 neurons) for trials with saccadic choices toward the RF (dark gray) or in the opposite direction (light gray). (b) Cumulative distribution of directional selectivity in the main task separated by decision stage and monkey. (c,d) Results from the control task, same format as in (a,b)

As shown in **Fig. 4**, target selection responses were weaker at the F relative to the I step, whether this was the first or second step in the sequence (**Fig. 4a, b** vs. **Fig. 4c,d**). Target selection indices (**Fig. 4b** **,** the differences between preferred and null direction saccades, calculated 250–300 ms after target onset) were positive at all decision steps (Wilcoxon test; all p<0.02 relative to 0), but were significantly smaller at the F relative to the I step in each task and monkey (Wilcoxon paired test; main task: monkey 1, p<0.006, monkey 2, p<0.02; control task, monkey 1, p<0.004, monkey 2, p<0.0005). These differences were not due to spurious factors such as ceiling effects (firing was far below the neurons’ maximal firing rates; **Fig. S2**), or to sensorimotor factors (as target salience and saccade metrics were equivalent at all decision steps; saccade amplitude, latency and velocity, all p > 0.1 for the effect of step). Therefore, the differences shown in **Fig. 4** are likely to reflect differences in decision strategies at the two decision steps. Thus, the conflict between the immediate and the final reward and the frequent value reversals, may have produced a weaker target selection response at the F relative to the I step.

### Neural learning is higher at the F relative to the I steps

The next question we asked is whether, consistent with the monkeys’ choices (**Fig. 2**), LIP neurons show faster learning of target values at the different steps. To evaluate this idea, we selected trials that ended in an optimal choice, thereby ensuring that we track the neural responses to a constant target as they evolve during a block. We then used a linear regression analysis to fit trial-by-trial firing rates as a linear function of trial number during a block. We calculated the regression slopes separately for saccades directed toward and opposite the RF, obtaining two slope parameters that indicated whether firing rates changed (increased or decreased) across trials for each saccade direction. Finally we calculated the difference between the two direction-specific slope coefficients. This provided a learning index which, when positive, indicates an increase in target selectivity during a trial block. Finally, to examine the time course of the learning effects, we calculated the learning index in a sliding window (100 ms width, 1 ms step) spanning the decision interval.

**Figure 5:**
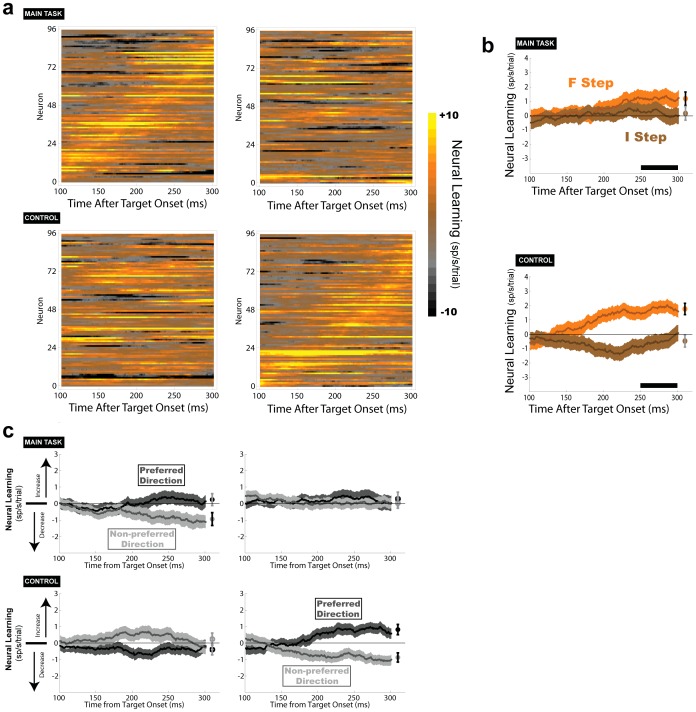
Neuronal learning is stronger at the F step. (a) Regression based analysis of neuronal learning. Each row in the colormap shows an individual neuron. Each pixel shows the learning index (color coded according to the scale on the right), computed in a sliding window that spans the delay period (100 ms time bins, 1 ms step). Within the main and control tasks, the neurons are sorted according to the time of their peak effect at the F step, so that corresponding rows show the same neuron at the two stages. (b) Population average learning indices (mean and SEM) across the neurons shown in (a). The symbols at the right edge summarize the statistical findings for comparisons 250–300 ms after target onset. Filled symbols indicate significant differences between the two traces (p<0.05). The color of the error bars indicate the result of comparison with 0, such that black shows p<0.05, and gray indicates p > 0.05. (c) Same as b, but with indices shown separately for the saccades directed toward the RF (dark gray) and in the opposite direction (light gray).

As seen from the colormaps in **Fig. 5a**, neuronal learning was significantly stronger at the F relative to the “I” step in both task contexts. This result was evident at the level of individual cells (**Fig. 5a**) and when the learning indices were averaged across the population (**Fig. 5b**), and was significant individually in each monkey (**Fig. S5**). We ruled out several possible artifactual explanations for these differences. Analysis of the directional slopes (**Fig. 5c**) showed that learning was due both to increases in firing for saccades to the RF and decreases in firing for saccades directed away, ruling out that it reflected a non-specific excitability change. Second, while the learning effects peaked at different times for individual cells (**Fig. 5a**) there were no correlations between the timing of a cell’s peak effect and task performance, task type or decision stage, or the time of the cell’s maximal selectivity for saccade direction. Third, the effects were not due to changes in firing variability, as there were no step-related differences in the across-trial variance or Fano factor (calculated separately for each cell and saccade direction in a sliding window throughout the delay period; all p > 0.1). Finally, the results were replicated in a separate analysis that compared target selection before and after the behavioral learn point (**Fig. S4**), showing that they were not artifacts of the analysis method. Therefore, these results indicate that value learning in LIP was consistently higher at the F relative to the I step regardless of the temporal order of the steps.

**Figure 6:**
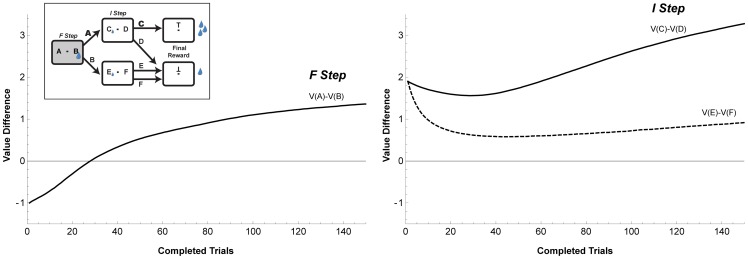
Predicted evolution of the value responses according to an RL simulation. Each panel shows the difference between the values of the optimal and non-optimal options, as a function of trial during a block, during the F (left panel) and I (right panel) of the main task. For the simulation we used a temporal discounting choice model, where the subjective value of each action (V) is updated by the temporally discounted prediction errors (R) resulting from both the immediate and the final rewards [12]. The simulations included all 5 task states (fixation, first step, re-fixation, second step, re-fixation with final reward), and calculated action values at each state according to the equation: 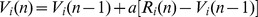 where V_i_(n) is the subjective value of action *i* after the *n*th time that action was selected, and α represents the learning rate. R is an internal estimate of the experienced reinforcement, specified by: 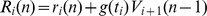 where *r* is the actual reward magnitude given at stage *i*, *g* is the hyperbolic temporal discounting function [7], and 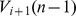 is the value of the next state before it is updated. Action selection was made using a ‘soft-max’ function, with a temperature parameter that introduced stochasticity in the monkeys’ choices. We did not fit the model to the data, but chose the model parameters so as to roughly replicate the monkeys’ behavioral pattern. However, we stress that the specific choice of the model parameters (learning rates, temporal discount and temperature) does not affect our argument, because all the parameters affect learning at both steps and produce the same qualitative pattern of results. This computational framework does not contain task-dependent learning control and thus by definition cannot produce state-specific learning allocation.

It is important to realize that, while these differentiated learning rates are consistent with the monkeys’ choices (**Fig. 2**) they are not a trivial consequence of the task setup or the monkeys’ choice pattern. To illustrate this point we simulated the monkeys’ performance on this task using a standard temporal difference algorithm, where learning was driven solely by temporally discounted prediction errors *without* a credit assignment mechanism (see legend to **Fig. 6** for the model details). We allowed the algorithm to converge across multiple reversals to simulate long-term experience with the two task contexts, and tested learning during a trial block after this long-term learning of the two contexts.

**Fig. 6** illustrates the evolution of action values during an AC-optimal block (the arguments apply equally to all configurations). As shown in **Fig. 6**, even though the algorithm replicated the monkeys’ *selective exploration* it produced *distributed value learning* at both decision steps. The monkeys’ stereotyped preference at the I step is explained by their long-term experience with the patterns at this step, which dictated that the relative value of target C would always be larger than that of target D (relative value estimates are positive at the I step from the start of the block). However, value learning did occur at the I step: the relative value of target C increased while that of target E decreased during the course of a block by an amount comparable to that seen at the first step (where relative values also changed sign). This is an obligatory consequence of the global final reward and the undifferentiated learning rule; learning at the I step is triggered simply by the fact that the final reward for target C is large an AC-optimal block but smaller in a BE-optimal block.

This simulation shows, therefore, that the selective learning in LIP cells unambiguously indicates a credit assignment mechanism. While the monkeys’ selective sampling strategy (**Fig. 2**) may have reflected overtraining with the task structure, the fact that neuronal (value) learning was stronger at the F relative to the I step even when the F step was relatively more distant from the final reward, requires a credit assignment mechanism and cannot be reproduced by a temporally dependent learning rule.

### Neurons encode the transition after the F step

**Figure 7:**
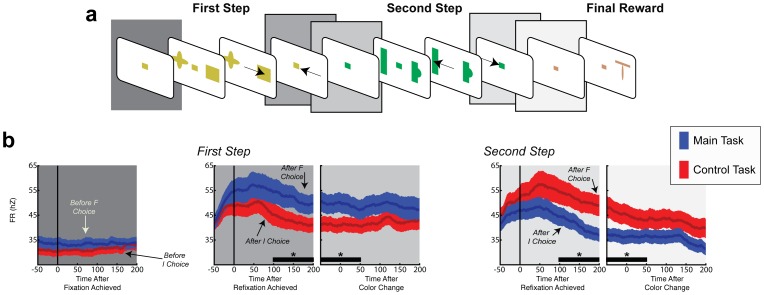
Non-spatial responses at state transition. (a) The sensorimotor events on a representative trial. The gray panels mark the events during state transitions – the return saccade to the fixation point and the change in fixation point color preceding the transition to the next state. During these periods of fixation, no targets were present and the monkey could not predict the saccade direction. (b) Non-spatial neural activity was enhanced after the F step. Average neural activity (n  =  96 cells) for the two task conditions aligned on the re-fixation saccade and change in fixation point color. Shading indicates SEM for each 1 ms time bin. The horizontal bars and stars denote a significant difference between the two traces (p<0.05).

We noted that, in addition to their step-specific target selection responses, LIP neurons had *post-decision* responses that were specifically elevated after F relative to the I steps. To illustrate this results, **Fig. 7** plots the population responses aligned on the post-decision events that link successive steps - the return to central fixation and the change in fixation point color heralding the transition to the following step (**Fig. 7a**).

During the main task, the neurons had higher firing after the *first* relative to the second step in the main task **(** **Fig. 7b** **, blue).** During the control task, in contrast, the neurons had higher firing after the *second* relative to the first step **(** **Fig. 7b** **, red)**. A 2-way ANOVA showed a significant effect of step type in the combined data and individually in each monkey (100–200 ms after saccade end: F  =  27.4, df(1,95), p<0.0001; p<0.03 monkey 1, p<0.0001 for monkey 2; 100 ms epoch centered on the change in the fixation point color: F  =  24.1, df(1,95), p<0.0001; p<0.05 for monkey 1, p<0.0001 for monkey 2). There was no significant task effect during the initial fixation (**Fig. 7b**, leftmost panel; F  =  1.8, df(1,95), p > 0.1), showing that the cells encoded a post-choice process rather than the preparation for the forthcoming choice. Post-decision responses were found in both pre-and post-learning trials, showing that they are independent of the monkeys’ knowledge of the optimal path. Finally, the responses were independent of the direction or reward of the preceding saccade, showing that they encoded to the preceding state rather than the action taken in that state (2 way ANOVA in the color-change aligned epoch; p > 0.08 for effect of saccade direction and direction x reward interaction for each task and decision stage; all p > 0.35 in paired comparisons of the two directions and two reward outcomes at each step and task). Thus, the cells had a non-spatial response, which was independent of the preceding reward or action and was highest during the transition after the F step.

## Discussion

We show that, during sequences of action oriented to a final reward, monkeys adjust their trial and error learning strategy according to the significance of a decision step, and these adjustments are reflected in differential value learning rates and post-decision responses in area LIP. We discuss the significance of these findings in light of previous studies of reinforcement learning and value representations.

Focusing primarily on single-step decision paradigms, studies of decision making have modeled the results of these paradigms using simple temporally dependent reinforcement learning rules. For instance, in a dynamic foraging task, Sugrue et al. found that the influence of a past reward on a current choice decays monotonically according to a memory time constant [6] and in a task of temporal discounting, Louie and Glimcher showed that the influence of an expected future reward decays monotonically with the expected delay [7]. Similarly, Bernacchia et al. showed that LIP neurons have reward learning across consecutive trials whose decisions are statistically unrelated, suggesting that learning is automatic and independent of the significance of an action [19].

Our results show that, in more complex sequential actions, learning in LIP has additional components that are temporally non-monotonic and based on the significance of individual actions. Value learning was enhanced at the F steps independently of time, indicating an augmented RL mechanism where trial and error learning is regulated by context dependent credit assignment mechanisms [9], [10]. As noted in computational studies, credit assignment is critical for efficient learning in complex conditions where agents cope with large option spaces [9] or perform several simultaneous tasks (e.g., walking while avoiding obstacles and picking up litter [10]). In such complex conditions credit assignment is critical for preventing inappropriate learning – i.e., assigning credit or blame to events that are irrelevant but happen to be close in time to the final outcome. Interestingly, in the control task LIP cells had slightly negative learning indices at the I step (**Fig. 5b**, bottom panel), suggesting a partial weakness of credit assignment, that leads to some unlearning of the optimal option.

LIP neurons showed two step-specific responses that are consistent with two mechanisms for credit assignment proposed in computational work. One mechanism is the selective increase in learning rates at the F relative to the I step, consistent with the theoretical proposal that credit assignment can be implemented by direct modulation of learning rates [10]. A second mechanism is the enhanced post-decision response after the F step, which may be related to eligibility traces that prolong the time for which an action is subject to reinforcement [11], [13], [20]. Note that the post-decision responses we describe are distinct from previously described memory traces in that they encoded the preceding state rather than the action or reward obtained in that state [21]. Most importantly, our post-decision responses encoded the specific reward-relevant step, rather than the mere time discounted value of relevant or irrelevant steps [7], [13], [21], [22].

As noted in the earlier sections, the transition diagrams defining the two contexts were not explicitly taught to the monkeys but had to be inferred **(** **Fig. 1**). Current research suggests two possible bases for these inferences. One possibility is that the monkeys inferred the underlying models – i.e., the transition contingencies – between successive steps, consistent with a growing literature showing that animals can flexibly modulate their exploration as if based on task models [23]. A second possibility is that the monkeys allocated learning based on the estimated uncertainty at each decision step, consistent with behavioral evidence that learning rates depend on context or state uncertainty [25], [26] [27], [28], [29], [30]. In the present task, uncertainty may have been signaled by the magnitude of the target selection response, which was smaller at the F relative to the I step, possibly indicating lower choice confidence at the former step [24]. Thus, our findings may be the first single-cell correlate of the selective learning described in behavioral investigations. It is important to note that both of these explanations - based on the transition structure and decision uncertainty – rely on *second-order* task properties, which must have been inferred through practice with a task or context. Therefore, our results require a hierarchical learning system that infers higher order task properties on a longer time-scale (i.e., inferring contextual properties over the experiment lifetime), and uses these inferences to guide exploration strategy on a short scale (i.e., finding the optimal path in a trial block).

Value learning is thought to recruit a distributed network of cortical and subcortical structures, and it is likely that this was also the case in our task [3]. The learning of task models and uncertainty/confidence estimates are linked with the frontal lobes [31] and volatility-driven learning rates have been proposed to involve a noradrenaline-linked arousal system [26]. Therefore the selective learning we find in LIP may reflect a distributed process, whereby model-based or uncertainty-based signals from the frontal lobe control the release of modulators at significant decision steps [32], [33], resulting in accelerated learning and cognitive allocation specifically at these steps.

## Methods

### Ethics statement

Two adult male rhesus monkeys (Macaca Mulatta) weighing 10–11 kg were tested with standard techniques [34]. This study was approved by the Animal Care and Use Committees of Columbia University and New York State Psychiatric Institute as complying with the guidelines within the Public Health Service Guide for the Care and Use of Laboratory Animals. Animals were pair-housed with compatible partners in cages of appropriate sizes, and were provided with environmental enrichment including foraging boards, toys, and daily interactions with trained human personnel. The animaĺs general health, appearance, and weight were monitored daily by experimenters and veterinary staff. Any sign of poor health (weight loss, diarrhea, shedding, etc.) was immediately addressed in consultation with the veterinarian. All behavioral training was done gradually, by personnel familiar to the monkey, using positive reinforcement of liquid or food rewards. The minimal necessary restraint was used for each procedure. Monkeys were allowed to drink to satiety during an experiment or were given supplemental water at the end of each session. On non-recording days monkeys were given as much water as on days when they work to satiation.

### General methods

During experimental sessions monkeys sat in a primate chair with their heads fixed in the straight-ahead position. Visual stimuli were displayed on a MS3400V XGA high definition monitor (CTX International, INC., City of Industry, CA; 62.5by 46.5 cm viewing area) at 57 cm in front of the monkeys’ eyes. The time of target presentation was measured with a photodiode detecting a vertical refresh.

### Identification of LIP

Electrode penetrations were aimed at the posterior half of lateral bank of the intraparietal sulcus as guided by structural MRI. Upon isolation, each neuron was tested with a memory-saccade task, where, after the monkey fixated a central point, a 1° diameter round target was flashed for 100 ms at a peripheral location. After a 1000–1250 ms delay, the monkey was rewarded for making a saccade to the remembered location of the target. All the neurons included in the study had spatial selectivity in the memory saccade task (1-way Kruskal-Wallis analysis of variance, p<0.05) and virtually all (99%) showed this selectivity during the delay or presaccadic epochs (200–900 ms after target onset and 200 ms before saccade onset). Median RF eccentricity in the neuronal sample was 11° (range, 8° – 14°).

### Sequential choice stimuli and task

the sequential decision task one choice target was presented in the RF and the second on the opposite side of fixation at an angular distance of at least 120° (typically 180°) relative to the first, with target locations randomized across trials. After each choice, the monkey returned to central fixation and viewed a change in the fixation point color heralding the progression to the next state. This sequence was repeated for the second decision and was followed by a small or large final reward, depending on the preceding choices. A delay of 300 ms was imposed between presentation of a target pair and the saccade go-signal (removal of the fixation point). Although longer delays are customary in single-choice tasks, shorter delays were necessary to improve performance given the long trial lengths in this task. Delays of 200-300 ms (200, 225, 250 or 300 ms chosen with uniform probability) were also imposed between each return to fixation and the change in color of the fixation point, between the change in color and the onset of the next pair of targets and between the final refixation and the final reward. Reward delivery was accompanied by presentation of an upright or inverted T signaling respectively, a large or small reward. Reward sizes differed across monkeys, being, typically, 0.003, 0.05 and 0.135 ml for monkey 1, and 0.007, 0.1 and 0.27 ml for monkey 2 (for, respectively, the immediate, small final and large final reward). Note that, despite any differences in absolute size, the rewards were related by a single scaling factor and thus did not alter the performance of individual monkeys. Moreover, the immediate rewards were very small, allowing the monkeys to orient their choices toward the final rewards.

The stimuli presented as saccade targets were abstract patterns distinguished by shape and color, subtending ∼1.8° on a side and approximately equated for luminance. Six stimuli were assigned to the main task and 6 to the control task, with two stimuli per stage for each configuration.

The task was presented in blocks of 100–150 trials, with the optimal path and conditions (main or control configuration) randomly interleaved across blocks. In 40 neurons (19 monkey 1, 21 monkey 2) we obtained two sets of trials for at least one task, thus studying both initial learning and reversal; since in these cases learning rates in both blocks were similar (p > 0.05, for each task), data from both blocks were pooled for analysis. As in other studies of learning, we found that monkeys had variable choice biases at the onset of a session, introducing noise in the baseline performance. To address this problem we preceded each session with a small number of “initializing” trials in which there was no inter-temporal conflict at the F stage – the same target led to the larger final and immediate reward. Monkeys quickly settled on choosing this target, and once this happened we began the actual task by switching the large final reward to the other target.

### Data analysis

Non-completed trials where monkeys broke fixation or made saccades away from the display were discarded and not analyzed further. Saccade latencies were determined offline using acceleration and velocity criteria. All neuronal analyses were conducted on raw firing rates. We analyzed firing rates between 200 - 300 ms after target onset – the later part of the decision period when choice effects are maximal. Note that on some trials this window extended beyond the disappearance of the fixation point, but this visual event was outside of the neurons’ RF and in all cases the window ended before the start of the saccade. Thus, this analysis window is not contaminated by visual or saccade artifacts. For display purposes only, response histograms were smoothed using a half-Gaussian kernel with a standard deviation of 20 ms. Analyses were preceded by normality and symmetry tests and, depending on the outcome, were based either on ANOVAs or paired-sample t-tests, or on non-parametric statistics. For the regression analysis of learning rates, trial-by-trial firing rates were normalized by subtracting the mean.

## Supporting Information

Figure S1 **Learning is gradual in individual sessions** To rule out the possibility that the gradual learning in the average data shown in **Fig. 2** is an averaging artifact, we plotted the performance in individual sessions. The figure shows the cumulative number of optimal choices as a function of trial number, drawn up to the learning point for each recording session in each task and monkey. In this representation an abrupt strategy shift would be seen as a line that is initially flat (indicating 0 optimal choices) but abruptly acquires a slope of 1 just before its end (before the learning point). Instead, monkeys showed a gradual accumulation of optimal choices, where streaks of optimal and non-optimal choices were interleaved (seen as interleaved sloped and flat line segments in this representation). This indicates that session-by-session learning was gradual, with no evidence of discrete shifts between two over-learned paths.
(PDF)

Figure S2 **Neural responses on the memory guided saccade task** The traces show the average firing rates (n  =  96 cells from both monkeys) on the memory guided saccade task when the target was inside the RF (solid) and at the diametrically opposite location (dashed). The neurons had the response pattern expected from LIP, including a transient visual response (first 100 ms of target presentation) and sustained spatially specific activity during the delay interval lasting up to 1,350 ms after target onset.
(PDF)

Figure S3 **LIP neurons independently encode value and saccade direction** Regression coefficients measuring sensitivity to value and saccade direction plotted for each monkey and task. The format is identical to main Fig. 4.
(PDF)

Figure S4 **Neuronal learning is stronger at the F step. (a)** Average directional selectivity (difference between preferred and null-direction saccades, mean and SEM), for trials ending in an optimal choice before and after the learn point. Background shading indicates the F step. **(b) Results from the control task,** in the same format as in (a). The asterisks denote a significant difference between the pre- and post-learning responses 200–300 ms after target onset (p<0.05). A 3-way ANOVA with factors of task type (main vs. control), decision step (F step vs. I step) and learning stage (pre vs. post-learn) showed a significant interaction such that learning was significantly stronger at the F step (F = 4.2, df(1,95), p<0.05). The lack of learning at the I step was not a ceiling effect, since neurons showed much stronger responses in the standard memory delayed saccade task (**Figure S3**).
(PDF)

Figure S5 **State-selective learning is robust in each monkey** The figure shows color maps of the neuronal learning effects, and a comparison of learning at the F and I step individually for each monkey. Conventions are identical to those in **Fig. 5a,b**, except that the color maps were rescaled for optimal visibility of the results in each monkey. Each monkey showed robust state-specific learning focused on the F step.
(PDF)
