# Supplementary figures and images for: Neural Correlates of Temporal Credit Assignment in the Parietal Lobe
Source: PLoS One. 2014 Feb 11;9(2):e88725. doi: 10.1371/journal.pone.0088725 (PMC3921206; doi:10.1371/journal.pone.0088725)

## MAIN TASK

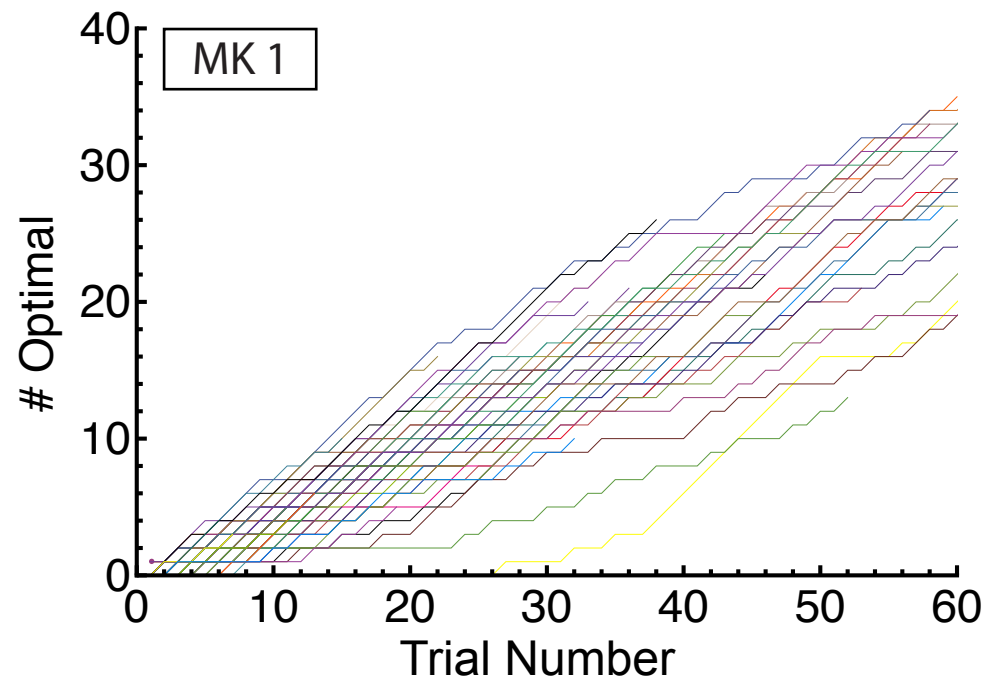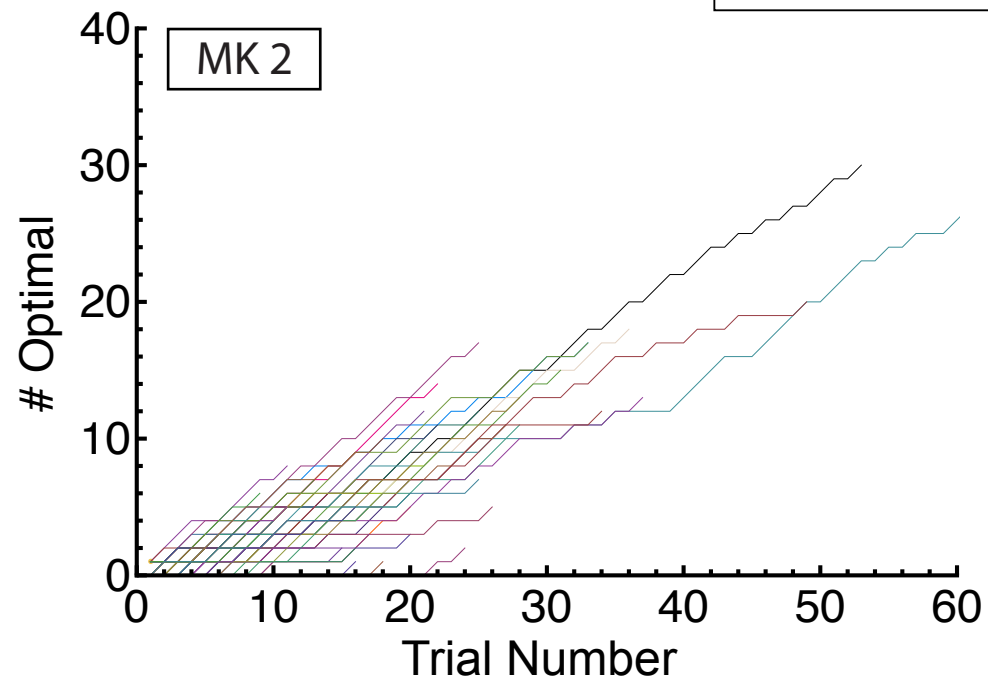

## CONTROL TASK

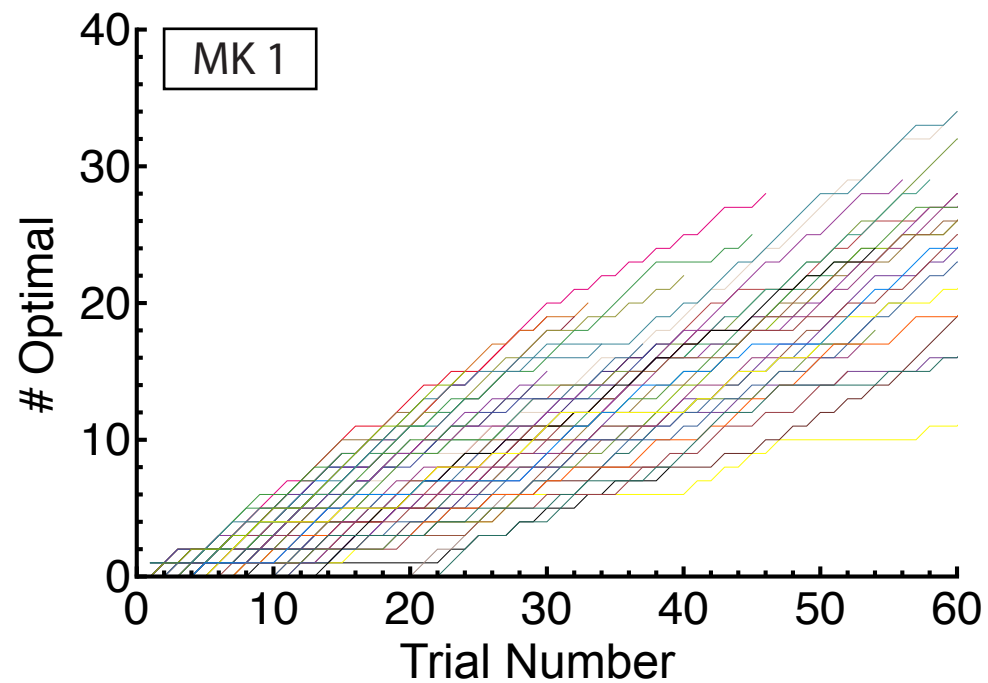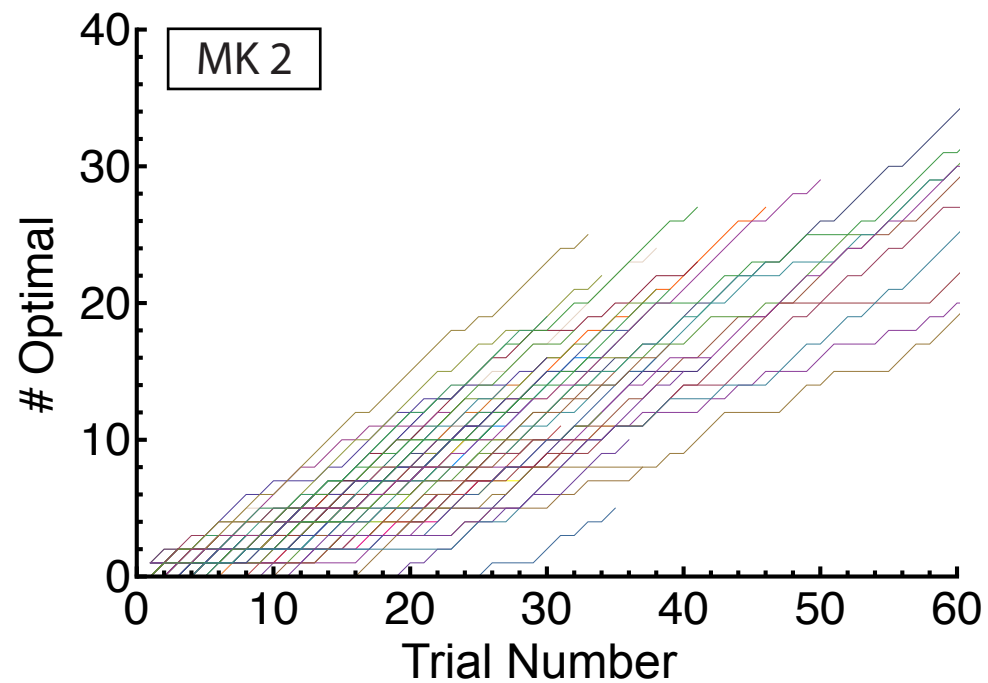

Figure S1

Supplement: Figure S1 — Learning is gradual in individual sessions To rule out the possibility that the gradual learning in the average data shown in Fig. 2 is an averaging artifact, we plotted the performance in individual sessions. The figure shows the cumulative number of optimal choices as a function of trial number, drawn up to the learning point for each recording session in each task and monkey. In this representation an abrupt strategy shift would be seen as a line that is initially flat (indicating 0 optimal choices) but abruptly acquires a slope of 1 just before its end (before the learning point). Instead, monkeys showed a gradual accumulation of optimal choices, where streaks of optimal and non-optimal choices were interleaved (seen as interleaved sloped and flat line segments in this representation). This indicates that session-by-session learning was gradual, with no evidence of discrete shifts between two over-learned paths. (PDF) [file pone.0088725.s001.pdf]

**Figure S2**

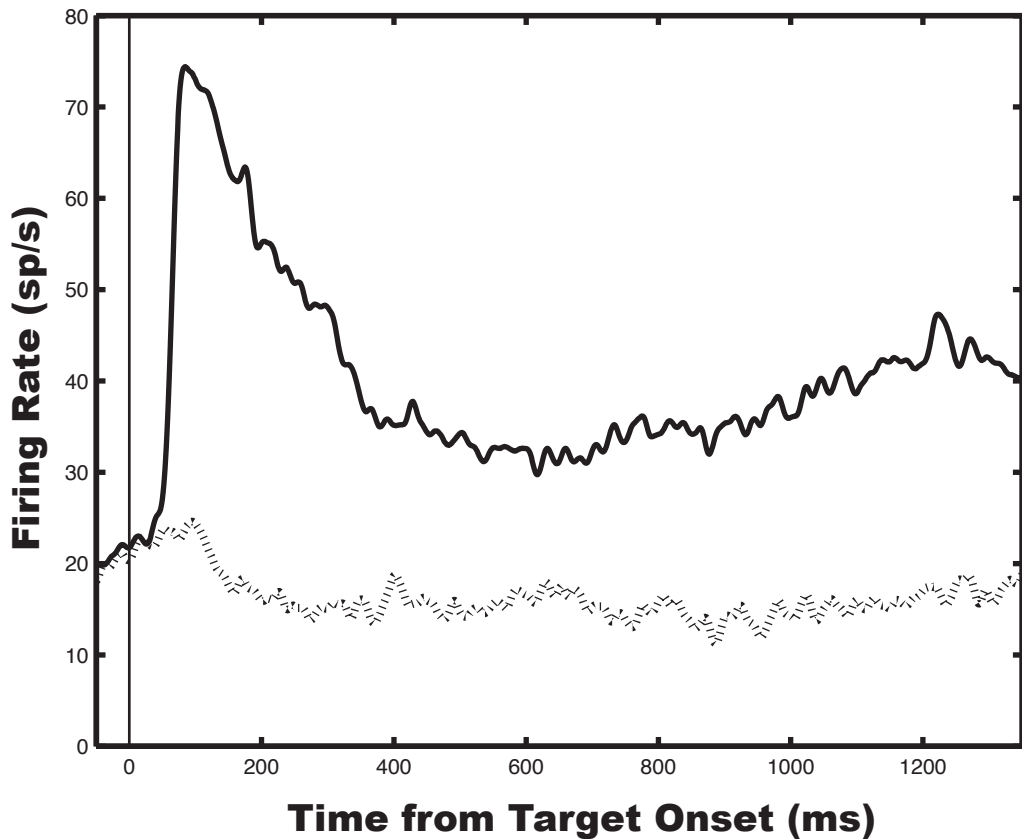

Supplement: Figure S2 — Neural responses on the memory guided saccade task The traces show the average firing rates (n = 96 cells from both monkeys) on the memory guided saccade task when the target was inside the RF (solid) and at the diametrically opposite location (dashed). The neurons had the response pattern expected from LIP, including a transient visual response (first 100 ms of target presentation) and sustained spatially specific activity during the delay interval lasting up to 1,350 ms after target onset. (PDF) [file pone.0088725.s002.pdf]

**Figure S3**

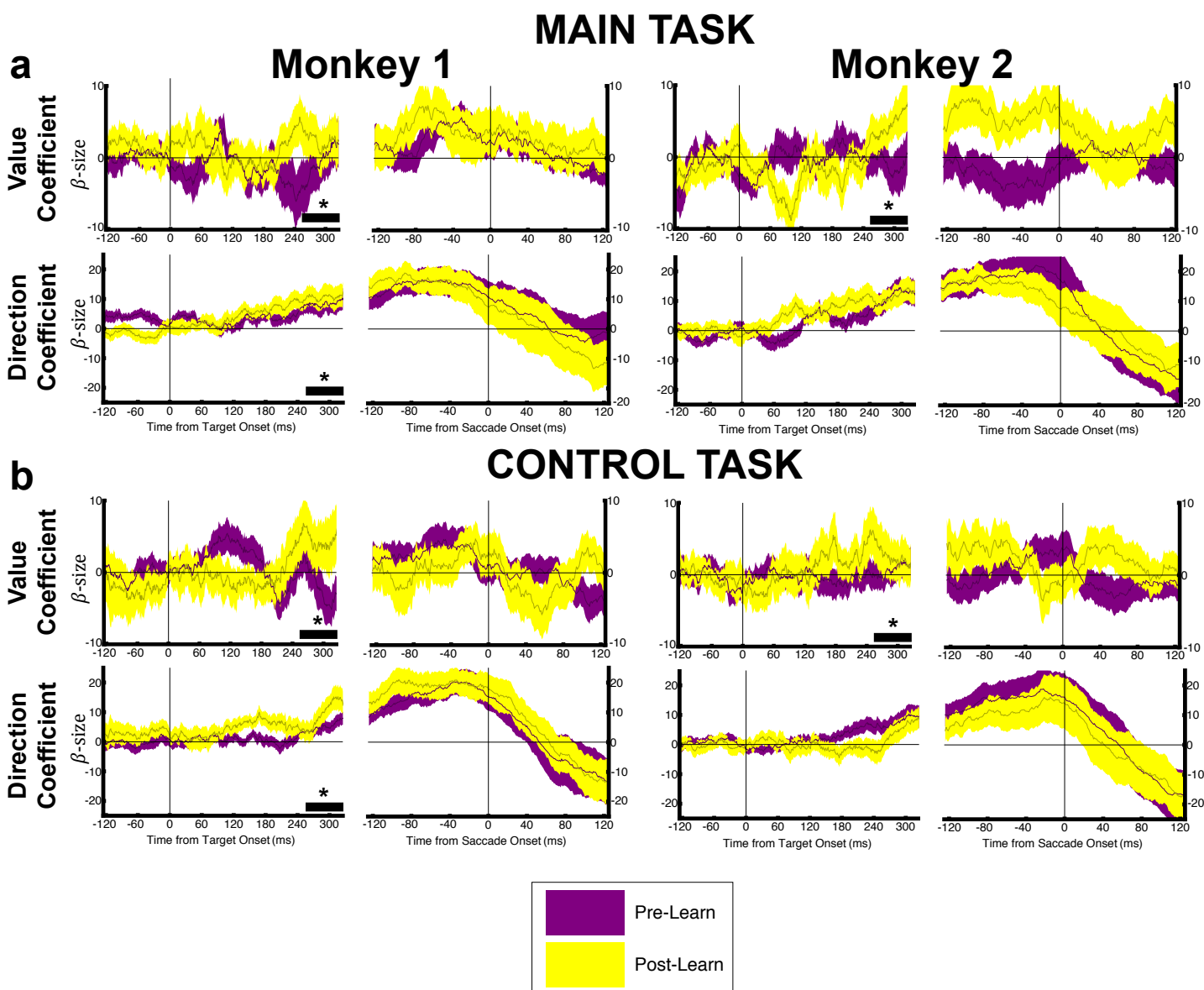

Supplement: Figure S3 — LIP neurons independently encode value and saccade direction Regression coefficients measuring sensitivity to value and saccade direction plotted for each monkey and task. The format is identical to main Fig. 4. (PDF) [file pone.0088725.s003.pdf]

**Figure S4**

**a**

**Main Task**

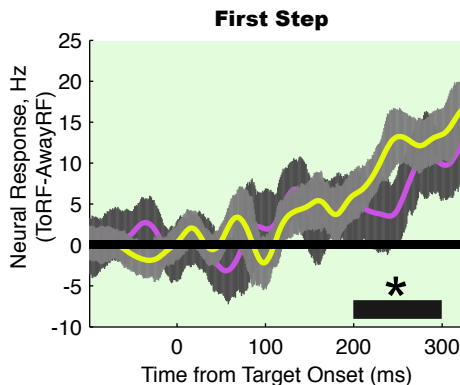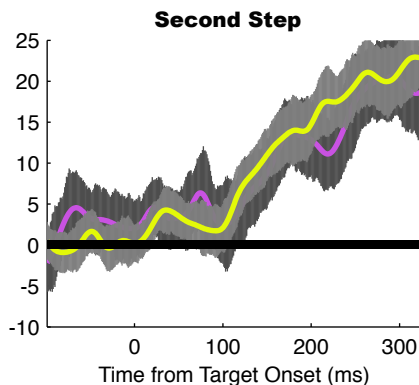

**b**

**Control Task**

— Pre-Learning

— Post-Learning

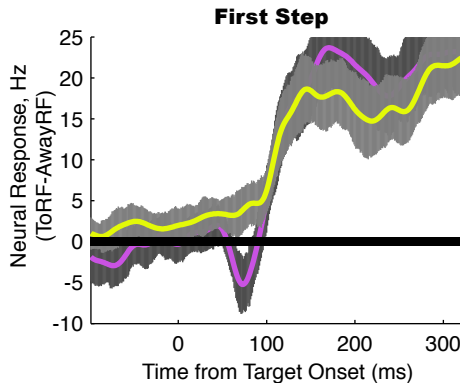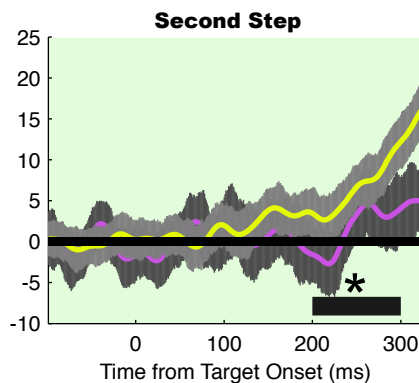

Supplement: Figure S4 — Neuronal learning is stronger at the F step. (a) Average directional selectivity (difference between preferred and null-direction saccades, mean and SEM), for trials ending in an optimal choice before and after the learn point. Background shading indicates the F step. (b) Results from the control task, in the same format as in (a). The asterisks denote a significant difference between the pre- and post-learning responses 200–300 ms after target onset (p<0.05). A 3-way ANOVA with factors of task type (main vs. control), decision step (F step vs. I step) and learning stage (pre vs. post-learn) showed a significant interaction such that learning was significantly stronger at the F step (F = 4.2, df(1,95), p<0.05). The lack of learning at the I step was not a ceiling effect, since neurons showed much stronger responses in the standard memory delayed saccade task (Figure S3). (PDF) [file pone.0088725.s004.pdf]

**a**

MK 1

**MAIN TASK**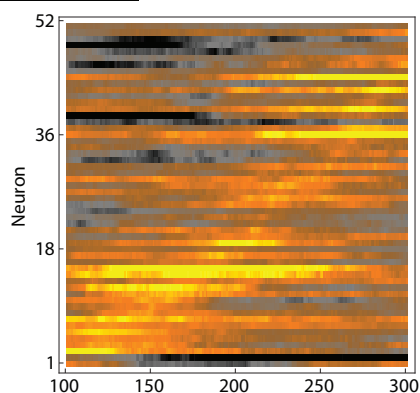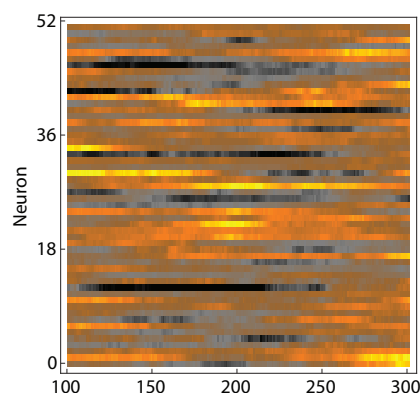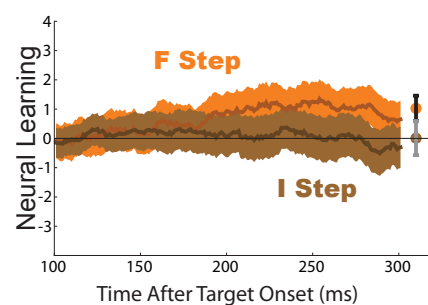**CONTROL**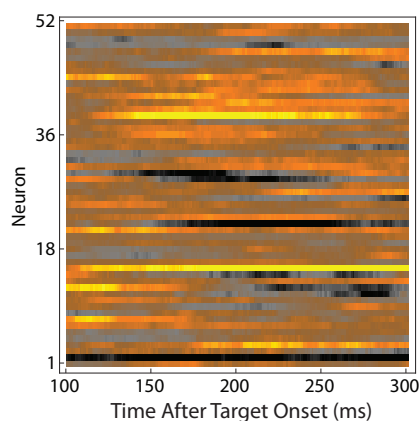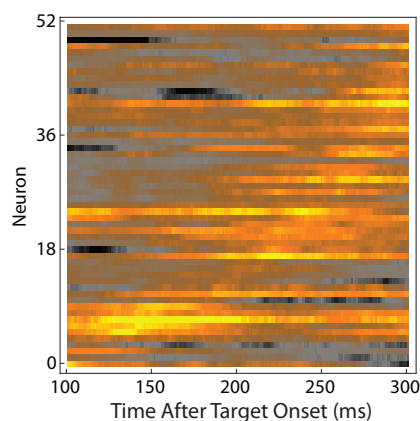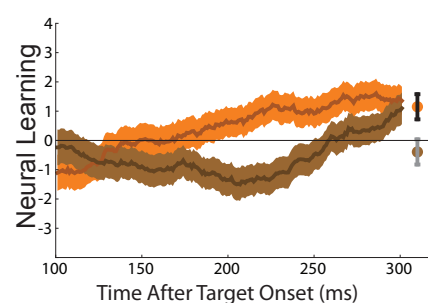Neural Learning ( $\Delta$  Slope)

-10

+10

**b**

MK 2

**MAIN TASK**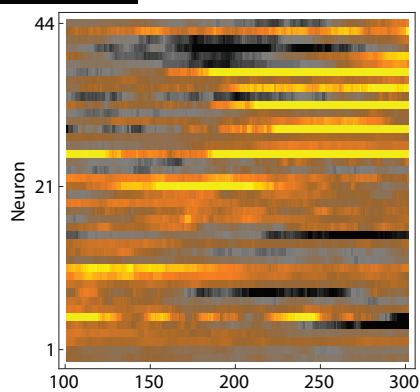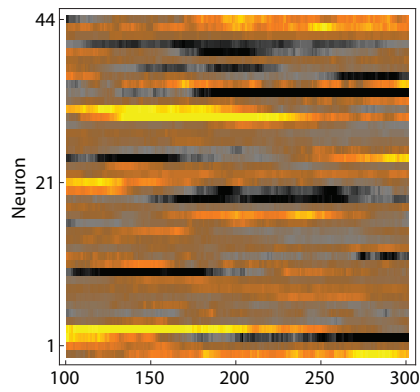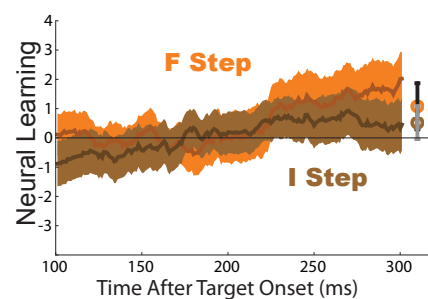**CONTROL**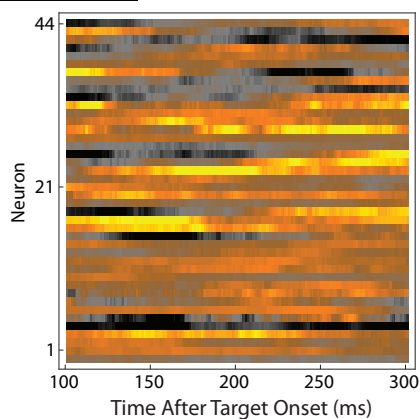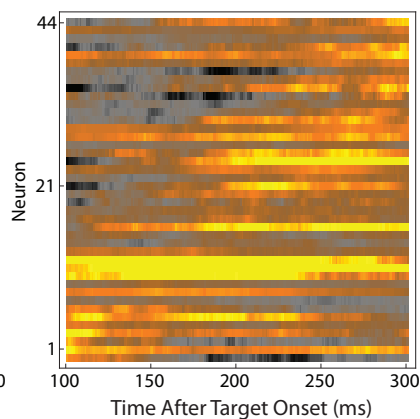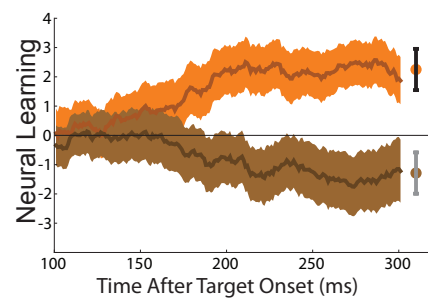

Supplement: Figure S5 — State-selective learning is robust in each monkey The figure shows color maps of the neuronal learning effects, and a comparison of learning at the F and I step individually for each monkey. Conventions are identical to those in Fig. 5a,b , except that the color maps were rescaled for optimal visibility of the results in each monkey. Each monkey showed robust state-specific learning focused on the F step. (PDF) [file pone.0088725.s005.pdf]
